# Identification of Key Genes Associated with Progression and Prognosis of Bladder Cancer through Integrated Bioinformatics Analysis

**DOI:** 10.3390/cancers13235931

**Published:** 2021-11-25

**Authors:** Shiv Verma, Eswar Shankar, Spencer Lin, Vaibhav Singh, E. Ricky Chan, Shufen Cao, Pingfu Fu, Gregory T. MacLennan, Lee E. Ponsky, Sanjay Gupta

**Affiliations:** 1 Department of Urology, School of Medicine, Case Western Reserve University, Cleveland, OH 44106, USA; sxv304@case.edu (S.V.); exs334@case.edu (E.S.); syl35@case.edu (S.L.); Lee.Ponsky@uhhospitals.org (L.E.P.); 2 The Urology Institute, University Hospitals Cleveland Medical Center, Cleveland, OH 44106, USA; 3 Department of Inflammation and Immunity, Cleveland Clinic Foundation, Cleveland, OH 44195, USA; singhv2@ccf.org; 4 Department of Population and Quantitative Health Sciences, Case Western Reserve University, Cleveland, OH 44106, USA; erc6@case.edu (E.R.C.); sxc519@case.edu (S.C.); pxf16@case.edu (P.F.); 5 Department of Pathology, Case Western Reserve University, Cleveland, OH 44106, USA; gtm2@case.edu; 6 Department of Nutrition, Case Western Reserve University, Cleveland, OH 44106, USA; 7 Department of Pharmacology, Case Western Reserve University, Cleveland, OH 44106, USA; 8 Division of General Medical Sciences, Case Comprehensive Cancer Center, Cleveland, OH 44106, USA; 9 Department of Urology, Louis Stokes Cleveland Veterans Affairs Medical Center, Cleveland, OH 44106, USA

**Keywords:** non-muscle invasive bladder cancer, muscle invasive bladder cancer, biomarkers, bioinformatics, prognosis, prediction

## Abstract

**Simple Summary:**

Bladder cancer is a heterogeneous disease with high recurrence rates. The current prognostication depends on tumor stage and grade and there is a need for predictive biomarkers that can distinguish between progressive versus non-progressive disease. We have identified a 3-gene signature panel having prognostic value in bladder cancer, which could aid in clinical decision making.

**Abstract:**

Bladder cancer prognosis remains dismal due to lack of appropriate biomarkers that can predict its progression. The study aims to identify novel prognostic biomarkers associated with the progression of bladder cancer by utilizing three Gene Expression Omnibus (GEO) datasets to screen differentially expressed genes (DEGs). A total of 1516 DEGs were identified between non-muscle invasive and muscle invasive bladder cancer specimens. To identify genes of prognostic value, we performed gene ontology (GO) and Kyoto Encyclopedia of Genes and Genomes (KEGG) analysis. A total of seven genes, including *CDKN2A*, *CDC20*, *CTSV*, *FOXM1*, *MAGEA6*, *KRT23*, and *S100A9* were confirmed with strong prognostic values in bladder cancer and validated by qRT-PCR conducted in various human bladder cancer cells representing stage-specific disease progression. ULCAN, human protein atlas and The Cancer Genome Atlas datasets were used to confirm the predictive value of these genes in bladder cancer progression. Moreover, Kaplan–Meier analysis and Cox hazard ratio analysis were performed to determine the prognostic role of these genes. Univariate analysis performed on a validation set identified a 3-panel gene set viz. *CDKN2A*, *CTSV* and *FOXM1* with 95.5% sensitivity and 100% specificity in predicting bladder cancer progression. In summary, our study screened and confirmed a 3-panel biomarker that could accurately predict the progression and prognosis of bladder cancer.

## 1. Introduction

Bladder cancer is a heterogeneous disease with high prevalence and recurrence rates [1]. According to an estimate by the American Cancer Society, in the United States, approximately 84,000 new bladder cancer cases will be diagnosed and 17,000 deaths will occur in the year 2021 (cancer facts and figure, American Cancer Society). The diagnosis and treatment of bladder cancer is based on a sequence of clinical stage with two distinct entities that include a low-risk, non-muscle invasive variant (NMIBC) and a highly aggressive muscle invasive (MIBC) subtype [2]. At initial diagnosis, about 70–80% of NMIBC tumors are confined to the epithelium or sub-epithelial connective tissue [3]. These tumors are frequently managed by surgery and/or immunotherapy based on their risk of progression assessment. Meanwhile, the best option for patients with loco-regionally advanced (intermediate to high-risk) disease remains standard chemotherapy. However, these treatments often become refractory over time with a high recurrence rate of 50–70% over a 5-year period and subsequent progression to MIBC [4,5]. To date, there has been no reliable means of prediction of disease progression other than clinical decision making models, risk estimates tables and nomograms [6]. The utility of these existing prediction models are less accurate as these classifiers are unable to provide information on the underlying biological properties that likely drive tumor behavior. Therefore, exploring molecular pathways and gene regulatory networks that provide information about disease progression may provide a new approach to predict prognosis and improvements in the management of bladder cancer.

An integral part of understanding the molecular basis of bladder cancer progression includes analysis of differentially expressed genes in NMIBC (low-risk) versus MIBC (high-risk) phenotype. At the molecular level, alterations in genes result in unrestricted cell proliferation, reduced cell death, invasion, and metastasis [7]. Specific alterations in gene regulatory network could affect the biologic behavior of the tumor that may influence patient’s survival. In fact, patients with aggressive disease require careful supervision, particularly because the treatment and pathogenesis of MIBC and NMIBC differ and conventional histopathological evaluation is inadequate to precisely predict the behavior of high-risk NMIBC [8]. Thus, there is a clear need of predictive biomarkers that can differentiate progressive from non-progressive NMIBC. Analysis of The Cancer Genome Atlas (TCGA) identified a distinct gene expression profile between NMIBC and MIBC [9,10]; however, limited prognostic values of these genes established the requirement for new molecular markers of bladder cancer outcome.

To examine the prognostic significance of genes and designate them as potential biomarkers for bladder cancer, we conducted sequential analyses on high-throughput sequencing data obtained from three datasets (GSE154261, GSE57813, and GSE37317) of bladder cancer. In the first step, differentially expressed genes (DEGs) were identified that are common among the three databases and analyzed using ingenuity pathway analysis (IPA). In the second step, we used Metascape to explore the major pathways of DEG enrichment in bladder cancer [11]. In the third step, the protein interaction network between DEGs was generated using the STRING online tool and demonstrated employing the Cytoscape software. Next, the Gene Expression Pro-filing Interactive Analysis 2 (GEPIA2) [12], and human protein atlas datasets were utilized to explore DEGs and their association with bladder cancer prognosis. Lastly, we discover single gene and its key biological role using the UALCAN, cBioPortal, and STRING online tools.

In the analysis, we identified 1516 DEGs commonly associated with bladder cancer progression. The DEGs aligned with functional interactome analysis comprising of 40 independent knowledge database revealed overrepresentation of cell cycle and cell-cycle check point associated genes in bladder cancer. We further validated the subset of selected core genes using independent datasets from TCGA and GEO databases and explored their potential as prognostic biomarkers in clinical use.

## 2. Materials and Methods

### 2.1. Cell Culture

Bladder cancer RT4, J82, HT1197, and 253JB-V cells were grown in RPMI 1640 (Catalogue number SH30027.01, GE Healthcare, Marlborough, MA, USA) supplemented with 10% fetal bovine serum, 50 U/mL penicillin and 50 µg/mL streptomycin in 100-mm tissue culture plates at 37 °C in a humidified atmosphere (5% CO_2_). The transformed bladder epithelial Urosta cells were grown in recommended culture medium supplemented with growth factors. The absence of mycoplasma contamination was tested using PCR-based assay (Catalogue MP0025; Sigma–Aldrich, St. Louis, MO, USA).

### 2.2. Data Set

The transcriptomic gene expression datasets of bladder cancer consisting of non-muscle invasive and muscle invasive subtypes were downloaded from the publicly available NCBI GEO (GSE154261, GSE57813, and GSE37317). The GSE37317 dataset contains 8 non-muscle invasive and 11 muscle invasive bladder cancer specimens, whereas GSE154261 dataset consists of 99 non-muscle invasive T1 stage bladder cancer specimens. The GSE57813 dataset comprised of 9 non-muscle invasive and 12 invasive T1G3 bladder cancer that are at high-risk of progression to muscle-invasive cancer (Table 1).

### 2.3. Data Processing

The datasets were analyzed using GEO2R (http://www.ncbi.nlm.nih.gov/geo/geo2r/, accessed on 8 June 2021) and limma R packages (Boston, MA, USA). The DEGs between the NMIBC and MIBC group were analyzed by using the ingenuity pathway analysis (IPA) (Qiagen, Redwood City, CA, USA), Metascape and Cytoscape. Finally, the genes with adjusted *p* < 0.05 and log2 fold-change FC > 1 or < –1 were set as the cut-off criteria. P-values were corrected for false discovery rate (FDR) using the Benjamini–Hochberg (B–H) test. To further enhance the reliability of the analysis, the overlapping DEGs co-existed in all 3 GSE data files were identified using IPA.

### 2.4. Functional and Pathway Enrichment Analysis

Metascape web based portal (http://metascape.org, accessed on 8 June 2021) was used to perform the pathway enrichment analysis, and the gene network reconstruction in human with default parameters set (minimum overlap 3, minimum enrichment 1.5, *p*-value < 0.01). Pathway and functional enrichment analyses were performed using the KEGG pathway, GO, reactome gene set, and canonical pathway. Specifically, *p*-values were calculated based on accumulative hypergeometric distribution, q values were calculated using the Benjamini–Hochberg (B–H) procedure to account for multiple testing. Kappa scores were used as the similarity metric when performing hierarchical clustering on the enriched terms, and then sub-trees with similarity >0.3 were considered a cluster. The most statistically significant term within a cluster was chosen as the one representing the cluster. To further capture the relationship among terms, a subset of enriched term was selected and rendered as a network plot, where terms with similarity >0.3 are connected by edges.

### 2.5. Quantitative RT-PCR

Total RNA was isolated from four bladder cancer cell lines viz. RT4 (transitional cell papilloma), J82 (transitional cell carcinoma), HT1197 (bladder carcinoma), and 253JB-V (metastatic phenotype) representing different cancer stage and transformed bladder epithelial Urosta cells using RNeasy mini kit (Qiagen, Germantown, MD, USA). The isolated RNA was treated with RNase-free DNase (Qiagen) to ensure samples were free of DNA. The cDNA was synthesized using 1 µg total RNA using high yield cDNA synthesis kit (Applied Biosciences, Waltham, MA, USA) followed the protocol as per manufacturer’s instructions. The cDNA (2 μL) was used as the template for PCR to quantify the abundance of transcript using specific primers for genes viz. *CDC20*, *CDKN2A*, *CTSV*, *FOXM1*, *MAGEA6*, *KRT23*, and *S100A9* (Supplementary Table S1). As an internal control, GAPDH (NM_008084), and actin (NM_007393) were used in the reaction. The qRT-PCR data was analyzed using REST-2009 software (Qiagen, USA), 2000 times the data was randomized (iterations), and relative expression ratio was calculated using the formula ratio = (*E*_target_)^ΔCP^_target (control-sample)_/(*E*_ref_)^ΔCP^_ref (control-sample)_. All values are in the 95% confidence interval were greater than 1, the interval is consistent with the REST 2009 at *p*-value of 0.000, compared with the significance level of 0.001.

### 2.6. Identification of Prognostic Genes

Gene expression profiling interactive analysis 2 (GEPIA2) was used for analyzing the RNA sequencing expression data of 9736 tumors and 8587 normal samples from the TCGA, using a standard processing pipeline with LogFC cutoff of 1 and *p*-value cutoff 0.01 to generate boxplot. To identify genes that exhibited stage-specific expression may represent potential prognostic marker. Stage specific gene expression analysis identified a subset of 7 genes viz. *CDC20*, *CDKN2A*, *CTSV*, *FOXM1*, *MAGEA6*, *KRT23*, and *S100A9* which were significantly altered in MIBC, compared to NMIBC. To analyze the expression of these genes in different subsets based on tumor grade, UALCAN web-based tool was used.

### 2.7. Statistical Analysis

Overall survival (OS) was measured from the date of diagnosis to the date of death and censored at the date of last follow up for survivors. Progression free survival (PFS) was measured from the date of diagnosis to the date of disease progression or the date of death and censored at the date of last follow up for those alive without disease progression. The effects of seven gene expressions on tumor aggressiveness (NMIBC versus MIBC), OS and PFS were estimated using logistic regression and Cox regression, respectively. The effects of gene expressions on OS and PFS were also analyzed using Kaplan–Meier method with log-rank test. All tests were two-sided and *p*-value ≤ 0.05 was considered statistically significant.

## 3. Results

### 3.1. Identification of DEGs in NMIBC versus MIBC

**Figure 1:**
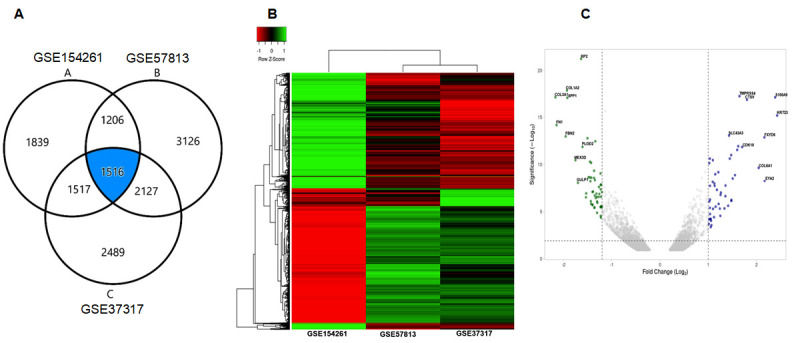
Identification of differentially expressed genes (DEGs) between 3 datasets. (**A**) Venn-diagram exhibiting three database GSE154261, GSE37317 and GSE57813 showing common DEGs associated with bladder cancer. (**B**) The composite heat map of three datasets. (**C**) The volcano plot of the three datasets.

We analyzed 3 NCBI-GEO database using IPA and aligned the DEGs with ingenuity knowledge database. A total of 1516 DEGs were identified, which were common from GSE154261, GSE57813, and GSE37317 databases (Supplementary Table S2) and processed with GEO2R (an interactive web tool) in order to identify genes that are differentially expressed (DEGs) with the adjusted *p*-value < 0.05 (Figure 1A). The data was analyzed and showed as a heat map for all 3 datasets and a volcano plot (Figure 1B,C).

### 3.2. GO and KEGG Pathway Analysis

**Figure 2:**
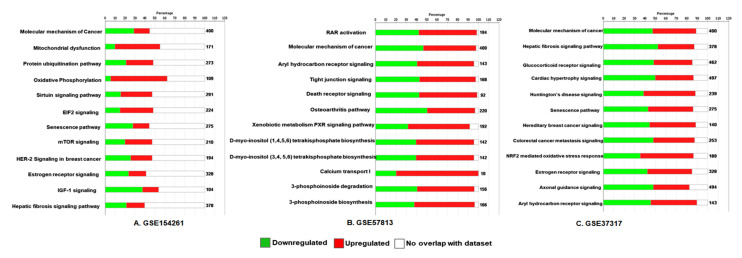
GO and KEGG pathway analysis of DEGs in bladder cancer. Bar diagram of three datasets (**A**) GSE154261, (**B**) GSE37317, and (**C**) GSE57813 analyzed for bladder cancer. Significant pathways with *p*-values < 0.01 were considered.

To investigate the prospective functions of DEGs, we performed GO analysis using Metascape online tools. The database aligned the DEGs with human genome database (GRCh38.p13), the bars represent the significant signaling pathway, which were discretely colored to encode p-values of increasing statistical significance. The analysis identified DEGs that were mainly enriched in biological pathways such as molecular mechanism of cancer, mitochondrial dysfunction, protein ubiquitination, oxidative phosphorylation, sirtuin signaling, EIF2 signaling, glucocorticoid receptor signaling, RAR activation, aryl hydrocarbon receptor signaling, tight junction signaling, and death receptor signaling pathways. KEGG pathway analysis revealed some highly ranked disease relevant pathways in all three databases, which were markedly enriched include hepatic fibrosis, cardiac hypertrophy, Huntington’s disease, osteoarthritis, and senescence pathways (Figure 2A–C).

**Figure 3:**
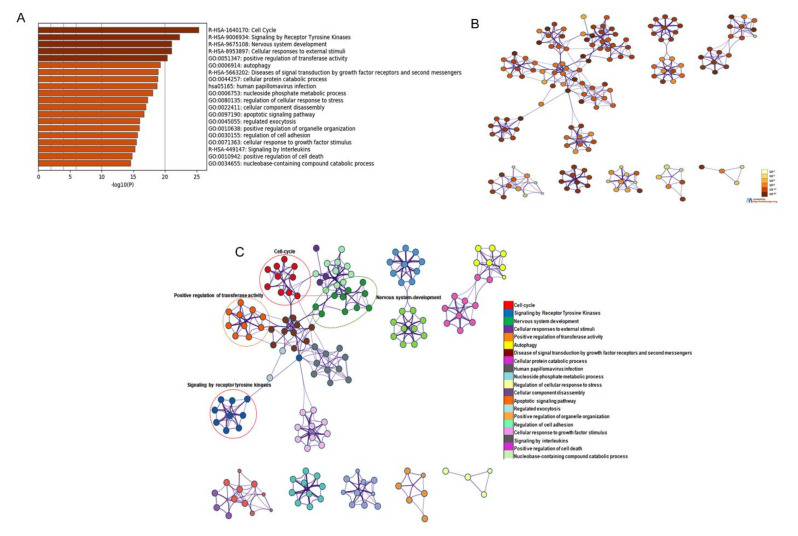
GO and KEGG pathway analysis of DEGs in bladder cancer from the Metascape website. (**A**) The results of KEGG pathway analysis were plugin Cytoscape software and discrete color scale to represent statistical significance in terms of *p*-value. (**B**) Colored by *p*-value, where terms containing more genes tend to have a more significant *p*-value. (**C**) Gene cluster enrichment network visualization generated using 1516 DEGs associated with bladder cancer. Each node represents one enrichment term and its color represents the individual cluster identity (nodes with same color belong to same cluster).

The analysis further identified that the DEGs are mostly enriched in cell-cycle signaling by receptor tyrosine kinase, nervous system development, cellular response to external stimuli, and others (Figure 3A–C).

### 3.3. Protein-Protein Interaction and Identification of Genes of Prognostic Significance

**Figure 4:**
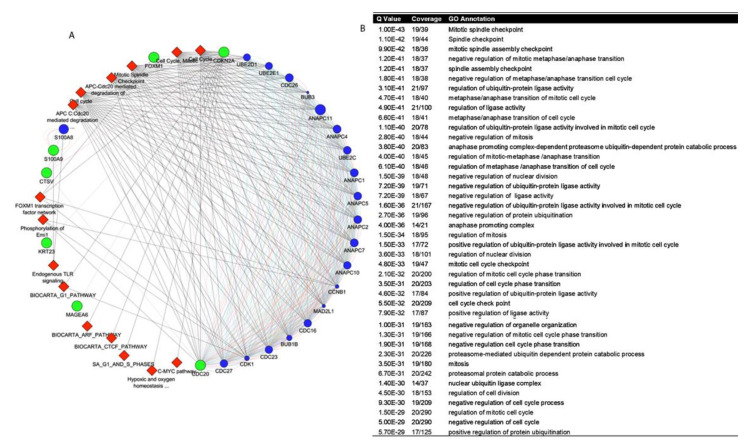
Determination of gene network in bladder cancer. (**A**) PPI network of 1516 overlapping target genes in bladder cancer. (**B**) GO annotation and z-score of the genes.

To further investigate the relationship between DEGs, the Cytoscape online application tool was applied (Figure 4A). We identified a subset of seven genes, which include *CDKN2A, CDC20*, *CTSV*, *FOXM1*, *KRT23*, *MAGEA6*, and *S100A9* (Table 2).

The query genes are presented in green, attributed gene network is represented in color blue, and associated pathways in red color. The result revealed the association of CDKN2A, CDC20, CTSV, FOXM1, KRT23, MAGEA6, and S100A9 with ANAPC11 (anaphase promoting complex subunit 11), S100A8 (S100 calcium binding protein A8), ANAPC7 (anaphase promoting complex subunit 7), ANAPC1 (anaphase promoting complex subunit 1), CD26 (cell division cycle 26), ANAPC4 (anaphase promoting complex subunit 4), and others are important in the regulation of cell cycle. Along with attributed genes, the cluster of genes showed its interaction with cell cycle, G1 pathway, G1 and S phases, mitotic spindle check point, c-MYC pathway and others as shown in red color (diamond shape). The Q-value, coverage and GO annotation are shown in Figure 4B. Furthermore, collagen type I and 3 alpha chain (COL1A2 and COL3A1), decorin (DCN), fibronectin (FN1), regulator of G protein signaling (RGS2), and secreted phosphoprotein (SPP1) were significantly downregulated in bladder cancer specimens, compared to noncancerous tissues (Supplementary Table S2).

### 3.4. Differential Expression of Selected Genes in Bladder Cancer and Normal Bladder Patients

**Figure 5:**
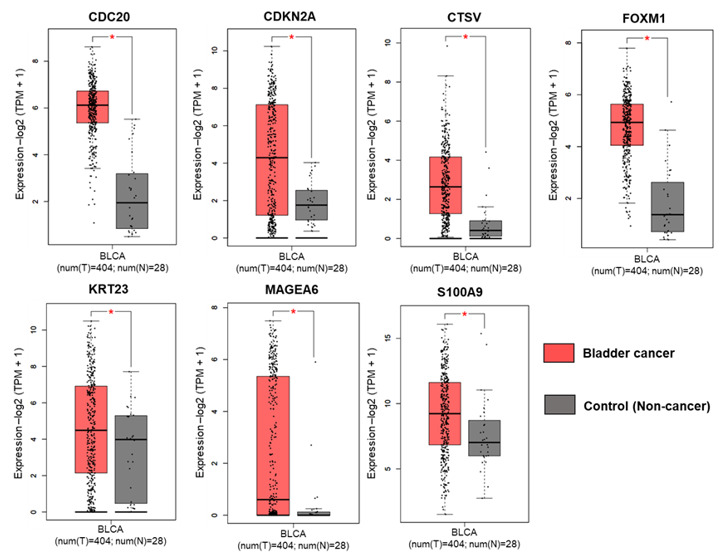
Expression analysis of seven gene subset in bladder cancer based on GEPIA. The expression of CDC20, CDKN2A, CTSV, FOXM1, KRT23, MAGEA6, and S100A9; * *p* < 0.05 was considered statistically significant.

To verify the differential gene expression of prognostic relevance, we analyzed the expression of seven genes between bladder cancer and normal bladder tissues. The subset of these genes were analyzed by using GEPIA2 within human patient cohorts consisting of bladder cancer (BLCA, n = 404) and normal bladder tissue (n = 28). The expression profile of CDC20, CDKN2A, CTSV, FOXM1, KRT23, MAGEA6, and S100A9 were significantly higher in bladder cancer specimens compared with normal bladder tissue in patients (Figure 5).

### 3.5. Stage-Specific Expression of Selected Genes in Bladder Cancer Progression

**Figure 6:**
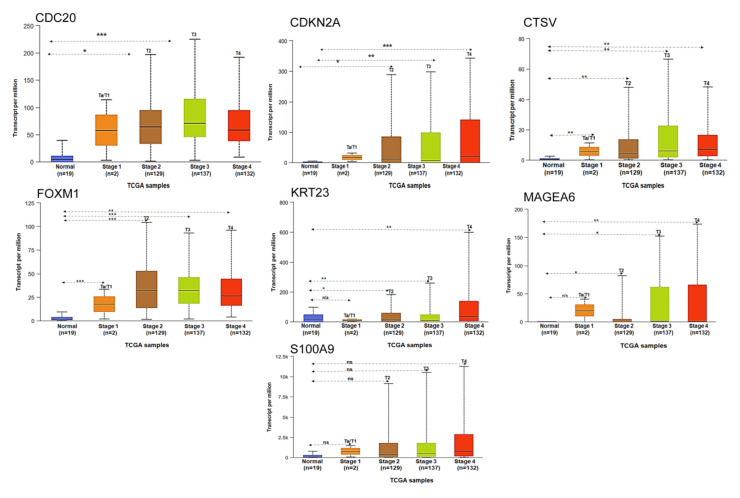
Expression of seven gene subset in bladder cancer based on sample types. The expression of CDC20, CDKN2A, CTSV, FOXM1, KRT23, MAGEA6, and S100A9 based on tumor stage. The figures were generated using ULCAN database. * *p* < 0.05, ** *p* < 0.001 and *** *p* < 0.0001 were considered statistically significant. ns, non-significant.

To analyze the stage-specific expression of seven subset of genes we used UALCAN –TCGA database. Within the seven subset of gene expression of CDC20, CDKN2A, CTSV, and FOXM1 significant changes in stage-specific expression were exhibited, while KRT23 and MAGEA6 showed changes at T2, T3, and T4 stages of bladder cancer, whereas no significant changes were noted between T1 and normal bladder tissue. S100A9 showed high level of expression at T2, T3, and T4 stages compared to Ta, T1 stages but the difference was non-significant (Figure 6). Log-rank P values showed statistical significance of the patterns observed between the groups.

**Figure 7:**
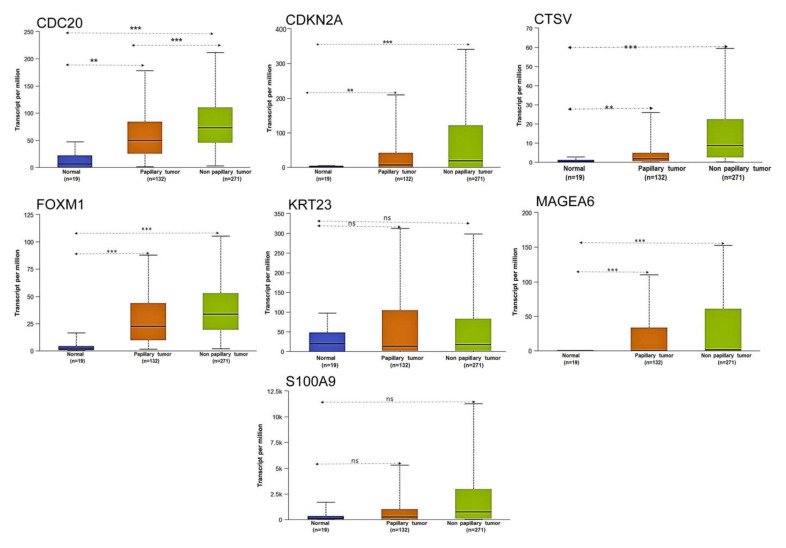
Expression of seven gene subset in bladder cancer based on histologic sample types. The expression of CDC20, CDKN2A, CTSV, FOXM1, KRT23, MAGEA6 and S100A9 based on tumor subtype. The figures are generated using ULCAN database. ** *p* < 0.001 and *** *p* < 0.0001 were considered statistically significant. ns, non-significant.

We also analyzed the seven gene subset and its expression based on histologic subtype. CDC20 and FOXM1 showed high level of expression in non-papillary tumors, compared to papillary tumors; the level of these proteins were significantly lower in normal bladder samples. While CDKN2A, CTSV, KRT23, and MAGEA6 showed significant differential expression in papillary and non-papillary tumors compared with normal tissue, no significant expression was observed between papillary and non-papillary tumors. Moreover, S100A9 showed significant changes in expression between papillary and non-papillary tumors but was not significant compared with normal tissue (Figure 7).

### 3.6. Survival Analysis of Selected Genes in Bladder Cancer

**Figure 8:**
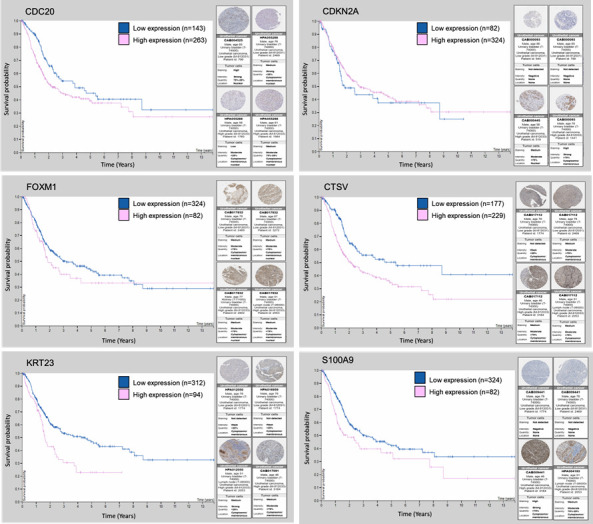
Kaplan–Meier survival plots of CDC20, CDKN2A, CTSV, FOXM1, KRT23, and S100A9 and their protein expression stratified into low-risk and high-risk groups from the TCGA and human protein atlas database.

We next examined the prognostic relevance of the gene subset in bladder cancer patient survival using the human protein atlas online tool for differential analysis. We found that higher expression of CDC20, CTSV, KRT23, and S100A9 correlate with poor survival, whereas lower expression of CDKN2A and FOXM1 were indicative of poor survival (Figure 8). The expression and survival analysis of MAGE6A was not shown in the protein atlas database.

### 3.7. Differential mRNA Expression of Selected Genes in NMIBC and MIBC Cell Lines

**Figure 9:**
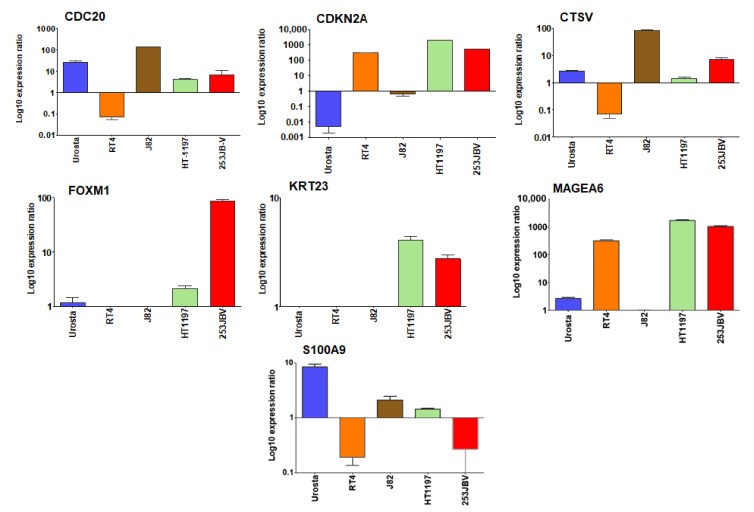
Validation of seven gene subset using qRT-PCR. Real time qRT-PCR validation of genes of CDC20, CDKN2A, CTSV, FOXM1, KRT23, MAGEA6 and S100A9. The qRT-PCR data was analyzed using REST 2009 © (Relative Expression Software Tool), Qiagen, Germantown, MD, USA. Bar represents the standard error mean (SEM) for three biologicals and three technical replicates.

In order to validate our findings, we determined the mRNA expression of seven DEGs selected for their prognostic significance. We selected four bladder cancer cell lines representing various stages of disease progression and transformed bladder epithelial Urosta cells, signifying as normal bladder epithelium. Compared to Urosta cells, CDC20 showed high expression (138.35 fold) in J82 cell line, a representative of epithelial transitional cell carcinoma line, and lower expression (3.98 and 8.47 fold) in HT1197 carcinoma cells and 253JB-V cells, which are metastatic variant at *p*-value 0.000. CDKN2A showed high level of expression (301.31, 2078.34, and 555.12 fold) in transitional papilloma RT4 cells, HT1197 cells, and 253JB-V cell line whereas J82 cells showed down-expression 0.5 fold at *p*-value 0.000. CTSV showed high level of expression (81.4 and 6.2 fold) in J82 and 253JB-V cell lines at *p*-value 0.000, and 0.069 fold downregulation in RT4 cell line at *p*-value 0.000. FOXM1 showed 1.85 and 86.13-fold overexpression in HT1197 and 253JB-V cell lines at *p*-value 0.047 and 0.000; whereas downregulation (0.023 and 0.25 fold) in RT4 and J82 cells at *p*-value 0.000. KRT23 showed similar trend of 3.85 and 2.42-fold higher expression in HT1197 and 253JB-V cells at *p*-value 0.039 and 0.002; whereas 0.1 and 0.021-fold downregulation in RT4 and J82 cells at *p*-value 0.004 and 0.000, respectively. Interestingly, MAGEA6 showed 317.25, 1707.45, and 1046.24-fold higher expression in RT4, HT1197, and 253JB-V cell lines at *p*-value 0.000. Expression of S100A9 was downregulated in all bladder cancer cells, compared to Urosta 0.16, 2.12, 1.45, and 0.13-fold downregulation in RT4, J82, HT1197, and 253JB-V cells at *p*-value 0.000 (Figure 9).

### 3.8. Prognostic Role of Selected Genes in Bladder Cancer

To analyze the prognostic significance of seven genes, tumor aggressiveness (NMIBC versus MIBC), overall survival (OS), and progression free survival (PFS) were estimated using logistic regression and Cox regression on an independent dataset. The patient characteristics are shown in Table 3.

**Figure 10:**
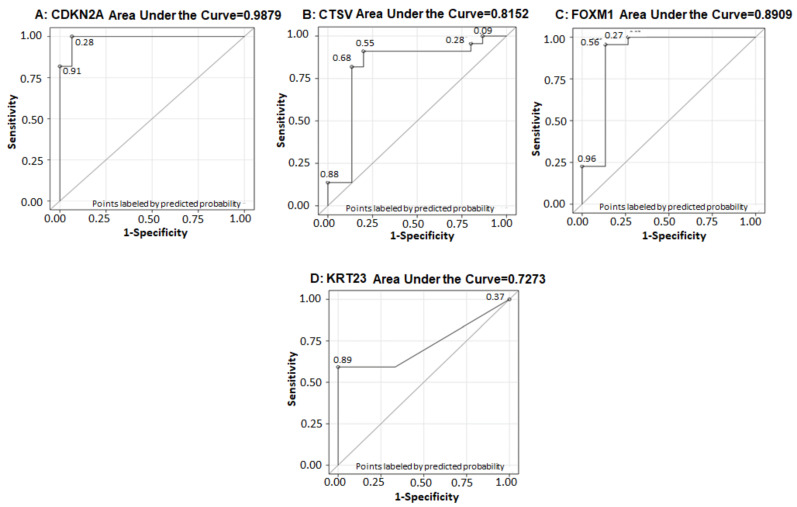
ROC curve for (**A**) CDKN2A, (**B**) CTSV, (**C**) FOXM1, and (**D**) KRT23 with AUC = 0.9879 for CDKN2A, AUC = 0.8152 for CTSV, AUC = 0.8909 for FOXM1, and AUC = 0.7273 for KRT23, respectively.

Univariate analysis was performed and the results demonstrated that receiver operative curve (ROC) for CDKN2A with AUC = 0.9879 (*p*-value < 0.01), sensitivity of 100% and specificity of 93.3% when the cutoff value of CDKN2a was 426.27 with a very good diagnostic performance (Figure 10A). The ROC curve for CTSV with AUC = 0.8152, sensitivity of 91% and specificity of 80% when the cutoff value of CTSV was 10.01, demonstrated good diagnostic performance (Figure 10B). A ROC curve analysis showed FOXM1 has very good diagnostic performance with AUC = 0.8909 and with the cutoff value of 104.67, the sensitivity and specificity for detecting tumor aggressiveness was 95% and 87%, respectively (Figure 10C).

The ROC curve for KRT23 with AUC = 0.7273, sensitivity of 59% and specificity of 100% when the cutoff value of KRT23 was 1.64, the analysis showed that KRT23 has not so good diagnostic performance with AUC of 0.7273 (Figure 10D); the p-values are shown in Table 4. The results of univariate analysis demonstrate that CDKN2A, CTSV, FOXM1 and KRT23 were predictive of tumor aggressiveness (MIBC) and the odds of having MIBC was increased by 3% per unit increase of CDKN2Aa (p = 0.01). The interpretation of other gene expressions remains similar. In addition, CDC20 values were predictive of OS (p = 0.014) and PFS (p = 0.021) the hazard of having disease progression or dying was increased by 12%. The univariate analysis of gene expression on OS and PFS is shown in Table 5 and Table 6.

### 3.9. Construction of Multiplex Model for Prognosis of Bladder Cancer

**Figure 11:**
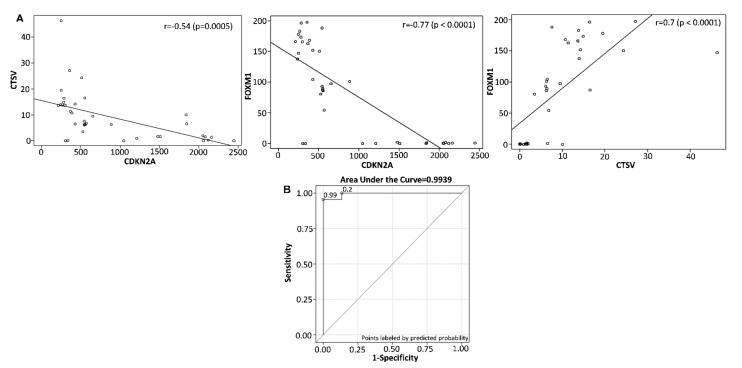
Scatter plot of (**A**) CDKN2A versus CTSV. The Pearson correlation coefficient between CDKN2A and CTSV was -0.54 (*p* = 0.0005). CDKN2A vs. FOXM1. The Pearson correlation coefficient between CDKN2A and FOXM1 was −0.77 (*p* < 0.0001). CTSV versus FOXM1. The Pearson correlation coefficient between CTSV and FOXM1 was 0.7 (*p* < 0.0001). (**B**) ROC curve for the combination of three biomarkers (CDKN2a, CTSV, and FOXM1) with AUC = 0.9939, sensitivity of 95.5% and specificity of 100%.

To evaluate if a multiplex model could improve performance over single biomarker(s), a three gene panel was selected, which demonstrated good diagnostic performance was subjected to determine the Pearson correlation coefficient. The Pearson correlation coefficient between CDKN2A and CTSV was −0.54 (*p* = 0.0005); CDKN2A and FOXM1 exhibited −0.77 (*p* < 0.0001); and CTSV and FOXM1 was 0.7 (*p* < 0.0001) (Figure 11A), demonstrating a variable selection to identify the minimum combination to accurately predict NMIBC progression. The ROC curve for the combination of a three gene set (CDKN2A, CTSV, and FOXM1) showed AUC = 0.9939. This analysis resulted in a final selection of a 3-gene model that contains CDKN2A, CTSV, and FOXM1. This model is capable of discriminating the progressive from non-progressive bladder cancer cases with sensitivity of 95.5% and specificity of 100% (Figure 11B).

## 4. Discussion

Predicting the risk of bladder cancer progression is often challenging because of histologic heterogeneity [13] and clinical characteristics of tumors. Therefore, there is a pressing need for identification of precise and novel biomarkers with prognostic significance. Our comprehensive analysis of databases identified 1516 differentially expressed genes (DEGs) between NMIBC and MIBC. The IPA analysis of individual dataset showed diverse overrepresented canonical pathways. In GSE154261 dataset, a total of 6078 DEGs were identified with significant enrichment in canonical pathways that include molecular mechanisms of cancer, mitochondrial dysfunction, protein ubiquitination pathway, oxidative phosphorylation, sirtuin signaling pathway, EIF2 signaling, senescence pathway, mTOR signaling, HER-2 signaling, estrogen receptor signaling, IGF-1 signaling, and hepatic fibrosis pathways. The GSE57813 dataset showed 7589 DEGs exhibiting RAR activation, molecular mechanism of cancer, aryl hydrocarbon receptor signaling, tight junction signaling and death receptor signaling, osteoarthritic pathway, PXR pathway, D-myo-inositol tetrakisphosphate biosynthesis, calcium transport, 3-phosphoinoside degradation and biosynthesis as overrepresented pathways. In the GSE37317 dataset, 7975 DEGs were noted and the pathways significantly overrepresented were molecular mechanism of cancer, hepatic fibrosis, glucocorticoid receptor signaling, and cardiac hypertrophy signaling, Huntington disease signaling, senescence pathway, hereditary breast cancer signaling, colorectal cancer metastasis, NRF2-mediated oxidative stress response, estrogen receptor signaling, axonal guidance signaling, and aryl hydrocarbon receptor signaling. Furthermore, the GO annotation and KEGG pathway enrichment analysis revealed pathways enriched in cell cycle/cell cycle checkpoint, signaling by receptor tyrosine kinase, nervous system development, cellular response to external stimuli and regulation of transferase activity, and autophagy. Previous studied showed that dysregulation of cell cycle events were closely associated with cell growth, anabolism, and cell proliferation, which comprise major hallmarks of cancer [14]. Therefore, investigating these signaling networks and disease-specific pathways are important to understand bladder cancer progression.

Several previous studies involved only one dataset for analysis. Nonetheless, a recent study by Xu et al. [15] analyzed three databases, while the present study collected more public dataset for analysis. We focused on transcript levels of DEGs in low-risk NMIBC and high-risk MIBC tissues. To explore the difference between the non-invasive versus invasive group, we identified the DEGs and analyzed by GO and KEGG pathway enrichment analysis. The enrichment analysis was constructed to demonstrate the nexus among key signaling pathways. It appears that the risk scores were predominantly related to the regulation of cell cycle. Interestingly the cell-cycle signaling pathway showed its close liaison with nervous system development, and positive regulation of transferase activity. Furthermore, the PPI network identified, mitotic spindle checkpoint, involvement of APC-CDC20, FOXM1 transcription factor network, phosphorylation of Emi-1, endogenous TLR signaling, CFCF, ARF, and c-MYC pathways, hypoxia and oxygen homeostasis as some key differentially expressed pathways. Besides, our analysis highlighted several attributed genes and their associated signaling network involved in bladder cancer prognosis, including the family of ubiquitin-conjugating enzymes (*UBE2C*, *UBE2D1*, and *UBE2E1*), regulatory protein involved in mitosis (*CCNB1*), cell division cycle protein ubiquitin ligase (*CDC16*, *CDC23*, and *CDC26*) that are components of the multiprotein APC complex. Other proteins involved in cell cycle regulation are the subunits of the anaphase-promoting complex (*ANAPC1*, *ANAPC2*, *ANAPC4*, *ANAPC5*, *ANAPC7*, *ANAPC10*, and *ANAPC11*), cyclin-dependent kinases (*CDK1* and *CDK27*), genes encoding kinases involved in the spindle checkpoint function (*BUB1B* and *BUB3*), mitotic spindle assembly checkpoint (*MAD2L1*) and S100 calcium and RAGE receptor binding protein (*S100A8*). The above data tempted to explore the gene regulatory network, which may give insight to understand its role in disease progression. The results of gene network analysis revealed the interaction of genes with anaphase-promoting complex/cyclosome (APC/C), which function together with one of homologous coactivators, CDC20. APC/C is an evolutionary conserved multi-subunit E3 ubiquitin ligase consisting of CDC16, CDC20, CDC23, CDC26, and CDC27 subunits that function to regulate progression of cells through the mitotic phase of the cell cycle. A defect in APC/C gene extends mitosis, inhibits drug-induced segregation errors in chromosomes, and diminishes naturally occurring lagging chromosomes in malignant cells. Indeed, somatic mutation in one the subunit CDC27 reduces chromosome segregation errors [16]. Additionally, it was demonstrated that the transcriptional regulation of CDC20 is mediated by FOXM1/transcription factor network, which is in accordance with our gene regulatory network data. BUB1B/MAD3L and BUB3 were also identified as molecules involved in the regulating gene network [17]. *BUB1B* gene encodes a kinase involved in spindle checkpoint function and plays a role in the inhibition APC/C by blocking the binding of *CDC20* to APC/C, delaying the onset of anaphase and ensuring proper chromosome segregation [18,19]. Regarding *CDKN2A* and its interaction with *MAGEA6* very little is known so far, moreover previous studies identified higher expression of MAGEA6 in early stage bladder cancer [20]. Cancer patients with high MAGEA6 expression exhibit poor prognosis. Conclusively, high expression of MAGEA6 in primary lesions could represent a promising prognostic biomarker [20,21].

The PPI network analysis of three databases identified the following subsets of DEGs, including *CDC20*, *CDKN2A*, *CTSV*, *FOXM1*, *KRT23*, *MAGEA6*, and *S100A9*, which were significantly upregulated in bladder cancer. Moreover, collagen type I and 3 alpha chain (*COL1A2* and *COL3A1*), decorin (*DCN*), fibronectin (*FN1*), regulator of G protein signaling (*RGS2*), and secreted phosphoprotein (*SPP1*) were significantly downregulated in NMIBC specimens, compared to MIBC tissues. In addition, some of the genes were in line with previously published studies [22,23,24]. The cell division cycle protein 20 (CDC20) homolog is a key regulator of cell division that is encoded by the CDC20 gene in humans that combines with the APC/C, and subsequently controls securin degradation. Loss of securin promotes the degradation of cohesion, interferes in sister chromatid segregation, and transition of cells from the G2/M phase to G1 phase of the cell cycle [25]. Abnormal expression of CDC20 promotes premature anaphase, resulting in mitotic anomalies and aneuploidy, which may promote carcinogenesis. Studies have demonstrated that CDC20 overexpression decreases overall survival and recurrence-free survival time in bladder cancer patients [22]. The molecular mechanism(s) of its overexpression is still unclear in bladder cancer, and further studies are required to establish it as a candidate biomarker.

CDKN2A, also referenced as cyclin-dependent kinase inhibitor 2A, encodes p16 protein that regulates cell cycle and is ubiquitously expressed in numerous tissues and cell types [26,27]. p16 inhibits CDK4 and CDK6 [28,29], the cyclin dependent kinases thereby activating the retinoblastoma (RB) family of proteins and its check on E2F transcription factors [30,31], which block progression of cells from G1 to S-phase of the cell cycle [32,33]. CDKN2A can activate tumor suppressor p53 either by binding and obstructing MDM2 protein to p53 or directly inhibiting RB protein during the G1-phase cell cycle [34,35]. Previous studies have demonstrated genomic alterations in CDKN2A in 20 to 60% of bladder carcinomas, which are associated with either RB1 deletions or E2F amplification [36,37]. Overexpression of CDKN2A is significantly observed in genomically unstable tumors associated with shorter progression-free survival in bladder cancer patients [38].

The CTSV gene encodes CTSL protein, which is a member of the peptidase C1 family, is a lysosomal cysteine proteinase that degrade extracellular matrix and basement membrane components such as type IV collagen, laminin, fibronectin, and proteoglycans of the bladder [24]. CTSL promoter activity and synthesis is driven by tumor secreted cytokines, and its upregulation is reported in a wide range of malignancies, including bladder cancer. High expression of CTSL correlates with metastatic aggressiveness and poor patient prognosis [39]. Evidence shows that CTSL expression may be linked to cancer grade and stage [40]. Studies have proposed CTSL as an independent predictor of bladder cancer and invasiveness in patients with a history of urothelial carcinoma [41,42].

FOXM1, a member of the forkhead gene family, is a regulator of embryogenesis and numerous developmental processes [43]. FOXM1 is primarily expressed in proliferating cells or induced by growth factor release. FOXM1 is designated as a proto-oncogene in most cancers [44], as it exclusively expressed in dividing cells and is involved in angiogenesis, cell migration and epithelial-to–mesenchymal transition. Relative research considers that FOXM1 plays an essential role in the phenotype determination and the development of molecular bladder cancer subtypes [45,46]. FOXM1 has been designated as a prognostic marker for bladder cancer and an independent predictor for overall survival and disease-specific survival in muscle-invasive bladder cancer [46]. The FOXM1 signaling network and its regulators, including FOXO3, PI3K, and AKT, remains putative drug targets in bladder cancer which requires additional work.

Human keratin 23 gene (KRT23) belongs to the family of type I keratins and 60–65% similarity within the alpha-helical rod domain [47]. Previous studies have reported abnormal expression of KRT23 in numerous tumor tissues, including bladder cancer. KRT23 is localized in the Golgi apparatus in the cytoplasm and is involved in the development and migration of various types of human cancers [48]. An association has been established between KRT23 and Smad4 in malignant cells, promoting Smad4-dependent upregulation and subsequent migration of cancer cells. However, the mechanism through which KRT23 regulates epithelial-to-mesenchymal transition in malignant cells remains unclear. Precise bio-informatics analysis provides evidence that KRT23 may upsurge epithelial-mesenchymal transition through regulation of TGF-β signaling pathway [49]. Additional studies are needed, especially in bladder cancer.

MAGEA6 (MAGE family member A6) is a protein coding gene associated with melanoma and osseous dysplasia [20]. MAGEA6 is overexpressed in a variety of human cancers, including bladder carcinoma [50]. Studies have shown that MAGEA6-TRIM28 ligase complex can degrade AMPK, a master cellular energy sensor and regulator, through ubiquitination of the alpha catalytic subunit (PRKAA1) resulting in overall reduction of AMPK protein levels in tumors [51]. Downregulation of AMPK by MAGEA6 led to significant decrease in autophagy and upregulation of mTOR, facilitating tumor growth. High MAGE6A expression is associated with poor clinical outcomes [52]. However, studies elucidating the tumorigenic role of MAGE6A in bladder cancer need further exploration.

S100A9 is a member of the low-molecular calcium binding S100 protein family implicated in regulating cell proliferation [53]. S100A9 is induced by various mitogens including VEGF-A, TGFβ and TNFα, increasing motility and promoting metastatic process. S100A9 supports proliferation and invasion of malignant cells which is dependent on receptor of advanced glycation end products (RAGE) and MAPK signaling cascades [54]. Studies have investigated the role of S100A9 in tumorigenesis [55]; however, the underlying molecular mechanism(s) in bladder cancer remains unknown.

We selected seven genes differentially regulated in NMIBC versus MIBC and their expression was analyzed in various bladder cancer cells representing stage-specific disease progression. Among the seven genes analyzed, *CDKN2A* and *MAGEA6* showed a progressive increase in the transcript with disease severity, whereas *CTSV*, *FOXM1* and *KRT23* gene transcript levels were higher in the carcinoma and metastatic carcinoma cell line. Irrespective of the tumor type, *CDC20* and *CTSV* showed higher expression in transitional cell carcinoma. Previous studies have shown abnormal expression of CDC20 in bladder cancer tissue that may serve as a potential diagnostic biomarker for NMIBC [56]. In addition, CDKN2A, is also designated as a molecular risk factor for tumor progression in NMIBC [38]. Our data suggest that overexpression of CDKN2A and MAGEA6 are associated with Ta/T1 stage NMIBC. The mRNA expression of seven genes were further confirmed from TCGA database demonstrating that the expression of these genes were markedly higher in bladder cancer compared with normal tissues. Indeed, the expression of CDC20, FOXM1, and MAGEA6 showed higher levels in tumors compared with normal tissues. Further, it will be interesting to validate the above set of genes in stage-specific manner in bladder cancer.

In order to validate the prognostic significance of the seven selected genes in low-risk NMIBC versus high-risk MIBC tumors, overall survival (OS) and progression free survival (PFS) were estimated using logistic and Cox regression. The results suggested that high expressions of these genes were associated with the malignant progression of bladder cancer. Furthermore, the mRNA expression levels of the subset genes were all significantly higher in MIBC tissues compared with NMIBC, which demonstrated that these seven genes played important roles in the progression of bladder cancer. The effect of expression of these genes were analyzed on survival and tumor aggressiveness in patients with bladder cancer. The univariate analysis of gene expression showed that CDC20 was predictive of OS (*p* = 0.014). Indeed, univariate analysis of gene expression on PFS using the Cox model showed the CDC20 was predictive of PFS (*p* = 0.021). Univariate analysis of gene expression data on tumor aggressiveness (NMIBC versus MIBC) using logistic regression showed genes, including *CDKN2A*, *CTSV*, *FOXM1*, and *KRT23*, were predictive of tumor aggressiveness.

The study has the following limitations and strengths. Firstly, there are a lack of in vivo experiments to validate the mechanisms of the genes in driving bladder cancer. Secondly, the sample size for validation analysis was small; therefore, some potential assays could not be performed. Lastly, NMIBC exhibit a heterogeneous subtype, thus multivariate analysis is needed to control the confounding effects, which requires substantial sample size. Therefore, further research with large sample size is warranted, which remains top priority on our research agenda. The strength of the study is the identification of a subset of seven genes, which are prognostic for bladder cancer. Furthermore, a combination of three gene set has 95.5% sensitivity and 100% specificity to differentiate between high-risk and low-risk disease subtype.

## 5. Conclusions

In summary, using a series of bioinformatics and retrospective analyses, the present study identified a subset of seven genes (*CDC20*, *CDKN2A*, *CTSV*, *FOXM1*, *KRT23*, *MAGEA6*, and *S100A9*), which were significantly associated with progression and prognosis of bladder cancer. Furthermore, the study also revealed a three-panel gene set (*CDKN2A*, *CTSV*, and *FOXM1*) that can accurately predict tumor progression and suggested as potential prognostic biomarker(s) for bladder cancer, which could aid in clinical decision making.

## Figures and Tables

**Table 1:** Information on GEO datasets and platforms.

GSE Dataset	Tumor Type	Tumor Type	DEGs	Platform
GSE154261	Non-muscle invasive (Surveillance) (*n* = 73)	Non-muscle invasive (Surgery) (*n* = 26)	6078	GPL20301 (Illumina HiSeq 4000) (Homo sapiens)
GSE57813	Non-muscle invasive (*n* = 9)	Muscle invasive (*n* = 12)	7975	GPL14951Illumina HumanHT-12 WG-DASL V4.0 R2 expression beadchip
GSE37317	Non-muscle invasive (n=8)	Muscle invasive (n=11)	7589	GPL96 [HG-U133A] Affymetrix Human Genome U133A Array

**Table 2:** Functional role and general information of the seven gene subset in bladder cancer.

Gene	Entrez Gene Name	Ensemble Gene ID	Molecule Type	Putative Biological Function	Location
CDKN2A	Cyclin Dependent Kinase Inhibitor 2A	ENSG00000147889	transcription regulator	Tumor suppressor	Nucleus
CDC20	Cell Division Cycle 20	ENSG00000117399	Enzyme	Cell cycle regulator	Nucleus
CTSV	Cathepsin V	ENSG00000136943	Peptidase	Lysosomal protease	Cytoplasm
FOXM1	Forkhead box M1	ENSG00000111206	Transcription regulator	Promotes oncogenesis	Nucleus
MAGEA6	MAGE Family Member A6	ENSG00000197172	other	Tumor progression	Cytoplasm
KRT23	Keratin 23	ENSG00000108244	other	Structural integrity	Cytoplasm
S100A9	S100 calcium binding protein A9	ENSG00000163220	other	Cell cycle progression	Cytoplasm

**Table 3:** Patient characteristics.

Factor	Frequency or Mean (STD) (n = 37)
Age (year)	65.9 (10.6)
Weight	82.1 (20.8)
Race (Asian/Black/White)	4/4/29
Stage (1/2/3/4)	2/13/12/10
Tumor aggressiveness (NMIBC / MIBC)	15/22
Tumor grade (High/Low)	34/3
Lymph node present (No/Yes)	5/30
Sex (female/male)	5/32

**Table 4:** Univariate analysis of gene expression data on tumor aggressiveness (NMIBC versus MIBC) using logistic regression.

Factors	Odds Ratio (95% CI)	*p* Value
CDKN2A (per unit increase)	1.03 (1.01, 1.05)	0.01
CDC20 (per unit increase)	1.08 (0.92, 1.25)	0.355
CTSV (per unit increase)	0.84 (0.74, 0.96)	0.008
FOXM1 (per unit increase)	0.97 (0.96, 0.99)	0.0009
KRT23 (per unit increase)	5.07 (1.32, 19.44)	0.018
MAGEA6 (per unit increase)	1.03 (0.97, 1.09)	0.419
S100A9 (per unit increase)	2.07 (0.63, 6.81)	0.233

**Table 5:** Univariate analysis of gene expression on overall survival using Cox model.

Factors	Hazard Ratio (95% CI)	*p* Value
CDKN2A (per unit increase)	0.999 (0.999, 1)	0.127
CDC20 (per unit increase)	1.12 (1.02, 1.23)	0.014
CTSV (per unit increase)	1.01 (0.96, 1.05)	0.747
FOXM1 (per unit increase)	1.004 (0.998, 1.01)	0.213
KRT23 (per unit increase)	1.19 (0.86, 1.64)	0.297
MAGEA6 (per unit increase)	1 (0.999, 1.001)	0.987
S100A9 (per unit increase)	0.94 (0.53, 1.66)	0.834

**Table 6:** Univariate analysis of gene expression on progression free survival using Cox model.

Factors	Hazard Ratio (95% CI)	*p* Value
CDKN2A (per unit increase)	0.999 (0.999, 1)	0.11
CDC20 (per unit increase)	1.12 (1.02, 1.22)	0.021
CTSV (per unit increase)	1.01 (0.98, 1.05)	0.529
FOXM1 (per unit increase)	1.002 (0.997, 1.01)	0.447
KRT23 (per unit increase)	1.08 (0.79, 1.47)	0.625
MAGEA6 (per unit increase)	1 (0.999, 1.001)	0.954
S100A9 (per unit increase)	1.27 (0.79, 2.06)	0.329

## Data Availability

The RNA-Seq data is available in NCBI-GEO database.

## Supplementary Materials

The following are available online at https://www.mdpi.com/article/10.3390/cancers13235931/s1, Table S1: List of Primers used for qRT-PCR, Table S2: Detailed list of differentially expressed genes from three datasets.
