# Supplementary material for: Identification of Key Genes Associated with Progression and Prognosis of Bladder Cancer through Integrated Bioinformatics Analysis
Source: Cancers (Basel). 2021 Nov 25;13(23):5931. doi: 10.3390/cancers13235931 (PMC8656554; doi:10.3390/cancers13235931)
Supplement: Supplementary file 1 [file cancers-13-05931-s001.zip › cancers-1373472/cancers-1373472-supplementary.pdf]

Table S1. List of Primers used for qRT-PCR

Genes	Sequence (5'…..3')
CDC20_qFor	CAAGGAGAACCAGCCTGAAA
CDC20_qRev	GGATCTTGGCTTCCTCTACATC
S100A9_qFor	GCTGGAACGCAACATAGAGA
S100A9_qRev	TCGCACCAGCTCTTTGAAT
MAGEA6_qFor	ACTACCATGAACTACCCTCTCT
MAGEA6_qRev	TACTGAGTGCTGCTTGGAAC
CDKN2A_qFor	ACGGGTCGGGTGAGAGT
CDKN2A_qRev	GGAAACTGGAAGCAAATGTAGGG
KRT23_qFor	GGTGACATCCACGAACTGAA
KRT23_qRev	AGACTGGGTCTCGGATAACA
FOXM1_qFor	GCAGCTAGGGATGTGAATCTT
FOXM1_qRev	AAGCCACTGGATGTTGGATAG
TAGLN_qFor	GTTCCAGACTGTTGACCTCTTT
TAGLN_qRev	CGGTAGTGCCCATCATTCTT
CTSV_qFor	AGCCTCTGTTTCTTGATCTTCC
CTSV_qRev	GAGCACCAGTCGCACTAAA
